# Supplementary material for: Heat adaptation of phage T7 under an extended genetic code
Source: Virus Evol. 2021 Dec 1;7(2):veab100. doi: 10.1093/ve/veab100 (PMC8923235; doi:10.1093/ve/veab100)
Supplement: veab100_Supp [file veab100_supp.zip › Sequencing_Analysis.zip!/Sequencing_Analysis/A1/evidence/RA_26.html]

BRESEQ :: Evidence


| Read alignment evidence... | | | | | | | | | | | |
| --- | --- | --- | --- | --- | --- | --- | --- | --- | --- | --- | --- |
|  | seq id | position | | ref | new | freq | score (cons/poly) | reads | annotation | genes | product |
| \* | NC\_001604.1 | 24,346 | 0 | C | T | 77.9% | 4251.2 / inf | 2870 | intergenic (–/–) | *–/–* | –/– |
| Reads supporting (aligned to +/- strand):  **ref** base C (300/326);  **new** base T (1084/1120);  **total** (1404/1466) | | | | | | | | | | | |
| Fisher's exact test for biased strand distribution *p*-value = 5.87e-01 | | | | | | | | | | | |
| Kolmogorov-Smirnov test that lower quality scores support variant *p*-value = 1.00e+00 | | | | | | | | | | | |

Only 100 of 2872 total aligned reads displayed.  

`GCGAGTGTTACTTCAACAGAAGAAACCTTAACGCCAGCACAGGAGGCCGCACGCACCCGCGCTGCTAACAAAGCCCGAAAGGAAGCTGAGTTGGCTGCTGCCACCGCTGAGCAATAACTAGCATAACCCCTTGGGGCCTCTAAACGGGTCTTGAGGGGTTTTTTGCTGAAAGGAGGAACTATATGCGCTCATACGATATGAACGTTGAGACTGCCGCTGAGTTATCAGCTGTGAACGACATTCTGGCGTCTATCGGTGAACCTCCGGTATCAACGCTGGAAGGTGACGCTAACGCAGATGCAGCGAACGCTCGGCGTATTCTCAACAAGATTAACCGACAGATTCAATCTCGTGGATGGACGTTCAACATTGAGGAAGGCATAACGCTACTACCTGATGTTTACTCCAACCTGATTGTATACAGTGACGACTATTTATCCCTAATGTCTACTTCCGGTCAATCCATCTACGTTAACCGAGGTGGCTATGTGTATGACCGAACGAGTCAATCAGACCGCTTTGACTCTGGTATTACTGTGAACATTATTCGTCTCCGCGACTACGATGAGATGCCTGAGTGCTTCCGTTACTGGATTG  >  NC_001604.1/24046‑24642`  

                                                                                                                                                                                                                                                                                                            `|`                                                                                                                                                                                                                                                                                                          
    `gagatgtgtataagagacagaCCTGAACGCCAGCACGGGAGGACGCACGCACCCGCGCTGCTAACAATGCCCGAAAGGAAGCTGAGTGGGCTGCTGCCACCGCTGAGCAATAACTAGCATAACCCCTTGGGGCATCTAAACGGGTCTTGAGGGGTTTTTTGCTGAAAGGAGGAACTATATGCGCTCATGCGATATGAACGTTGAGACTGCCGCTGTGTTATCAGCTGTGAACGACATTCTGGCGTCTATCGGTGAACCTCCGGTATCAACGCTGGAAGGTGACGCTAACGCAGATGCAGCg                                                                                                                                                                                                                                                                                                      <  1:339724/281‑1 (MQ=255)`  
       `ttACTTCAACAGAAGAAACCTTAACGCCAGCACAGGAGGCCGCACGCACCCGCGCTGCTAACAACGCCCGAAAGGAAGCTGAGTTGGCTGCTGCCACCGCTGAGCAATAACTAGCATAACCCCTTGGGGCCTCTAAACGGGTCTTGAGGGGTTTTTTGCTGAAAGGAGGAACTATATGCGCTCATACGATATGAACGTTGAGACTGCCGCTGAGTTATCAGCTGTGAACGACATTCTGGCGTCTATCGGTGAACCTCCGGTATCAACGCTGGAAGGTGACGCTAACGCAGATGTAGCGAAc                                                                                                                                                                                                                                                                                                   <  1:323077/301‑1 (MQ=255)`  
                    `agaaACCTTAACGCCAGCACAGGAGGCCGCACGCACCCGCGCTGCTAACAAAGCCCGAAAGGAAGCTGAGTTGGCTGCTGCCACCGCTGAGCAATAACTAGCATAACCCCTTGGGGCCTCTAAACGGGTCTTGAGGGATTTTTTGCTGAAAGGAGGAACTATATGCGCTCATGCGATATGAACGTTGAGACTGCCGCTGAGTTATCAGCTGTGAACGACATTCTGGCGTCTATCGGTGAACCTCCGGTATCAACGCTGGAAGATGACGCTAACGCAGATTCAGCGAACGCTCGGCGTAttt                                                                                                                                                                                                                                                                                      >  1:242158/1‑300 (MQ=255)`  
                      `aaaCCTTAACGCCAGCACAGGAGGCCGCACGCACCCGCGCTGCTAACAAAGCCCGAAAGGAAGCTGAGTTGGCTGCTGCCACCGCTGAGCAATAACTAGCATAACCCCTTGGGGCCTCTAAACGGGACTTGAGGGGTTTTTTGCTGAACCGAGGAACTATATGCCCTCATACGATATGAACGTTGAGACTGCAGCTGAGTTATCACCTGTGAACTACATTCTGTCGTCTATCGGTGAACCTCCGGTATCTACGCTGGATCGTTACCCTAACCCAGATGTAGCGAACGCTCGGCGTCTtctc                                                                                                                                                                                                                                                                                    >  1:23602/1‑301 (MQ=255)`  
                        `aCCTTAACGCCAGCACAGGAGGCCGCACGCACCCGCGCTGCTAACAACGCCCGAAAGGAAGCTGAGTTGGCTGCTGCCACCGCTGAGCAATAACTAGCATAACTCCTTGGGGCCTCTAACCGGGTCTTGAGGTGTTTTTTGCTGAAACGAGGAACTATATGCGCTCATACGATATGAACGTTGAGACTGCCGCTGAGTTATCAGCTGTGAACTACATTCTGGCGTCTATCGGTGAACCTACGGTATCAACGCTGGAAGGTGACGCTAACGCAGATGTAGCgctgtctcttatacacatctc                                                                                                                                                                                                                                                                                  >  1:169454/1‑281 (MQ=255)`  
                         `ccTTAACGCCAGCACAGGCGGCCGCACGCACCCGCGCTGCTAACAAAGCCCGAACGGAAGCTGAGTTGGCTGCTGCCACCGCTGAGCAATAACTAGCATAACCCCTTGGGGCCTCTAAACGGGTCTTGAGAGGTTTTTTGCTGAAAGGAGGAACTCTATGCGCTCATACGATATGAACGTTGTGCCTGCTCCTGCGCTATCAGCTGCGAACCACATTCTGGCGTCTATCGGTGAACCTCCGGTATCAACGCTGGAAGGTGACGTCAACGACGATGTAGCAGACGCACGGCTTAGTCTcaaa                                                                                                                                                                                                                                                                                 >  1:487462/1‑300 (MQ=255)`  
                              `agacaggCACAGGAGGCCGCACGCACCCGCGCTGCTAACAAAGCCCGAAAGGAAGGTGAGTTGGCTGCTGCCACCGCTGAGCAATAACTAGCATAACCCCTTGGGGCCTCTAAACTGGTCTTGATGGGTTTTTTGCTGAAAGGAGGAACTATATGCGCTCATACGATATGAACGTTGAGACTGCCGCTGAGTTATCAGCTGTGAACGACATTCTGGCGTCTATCGGTGAACCTCCGGTATCAACGCTTGAAGGTGACGCTAACGCAGATGCAGCGAACGCTCGGCGTATTCTCAACAAGAt                                                                                                                                                                                                                                                                            <  1:52641/295‑1 (MQ=255)`  
                                 `ccAGCACAGGAGGCCGCACGCACCCGCGCTGCTAACAAAGCCCGAAAGGAAGCTGAGTTGGCTGCTGCCACCGCTGAGCAATAACTAGCATAACCCCTTGGGGCCTCTAAACGGGTCTTGAGGGGTTTTTTGATGAAAGGAGGAACTATATGCGCTCATACGATATAAACGTTGAGACTGCCGCTGCGATATCAGCTGTGAACGACATTCTGTCGTCTATCGGTGAACCTCCGGTATCAACGCTGGAAGGTGACGCTAACGAAGATGTAGCGAACGCTCGGCGTATTATCAACAAGAttca                                                                                                                                                                                                                                                                         >  1:301734/1‑299 (MQ=255)`  
                                      `acaGGAGGCCGCACGCACCCGCGCTGCTAACAAAGCCCGAAAGGAAGCTGAGTTGGCTGCTGCCAGCGCTGAGCAATAACTAGCATAACCCCTTGGGGCCTCTAAACGGGTCTTGAGGGGTTTTTTGCTGAAAGGAGGAACTATATGCGCTCATACGATATGAACGTTGAGACTGCCGCTGAGTTATCAGCTGTGAACGACATTCTGGCGTCTATCGGTGAACCTCCGGTATCAACGCTGGAAGGTGACGCTAACGCAGATGTAGCGAACGCTCGGCGTATTCTCAACAAGATTAACCGAc                                                                                                                                                                                                                                                                    <  1:178571/301‑1 (MQ=255)`  
                                          `gaggCCGCACGCACCCGCGCTGCTAACAAAGCCCGAAAGGAAGCTGAGTTGTCTGCTGCCACCGCTGAGCAATAACTAGCATAACCCCTTGGGGCCTCTAAACGGGGCTTGAGGGGTTTTTTGCTGAAAGGAGGAACTATATGCGCTCATACGATATGAACGTTTAGACTGCCGCTGAGTTATCAGCTGTGAAATACATCCTGGCGTTTATCGTCTATCCTCCTGTATCAACGCTGGAAGGTGACGCTAACTCATATGTAGCGAACGCTCGGCTTCTTCTCACCCAGATTACCCgtccgtt                                                                                                                                                                                                                                                                >  1:334347/1‑295 (MQ=255)`  
                                           `aggCCGCACGCACCCGCGCTGCTAACAAAGCCCGAAAGGAAGCTGAGTTGGCTGCTGCCACCGCTGAGCAATAACTAGCATAACCCCTTGGGGCCTCTAAACGGGTCTTGAGGGGTTTTTTGCTGAATGGAGGAACTATATGCGCTCATGCGATATTAACGTTGAGACTGCCGCTGAGTTATCAGCTGTGAACGACATTCTTGCGTCTATCGGTGAACCTCCGGTATCAACGCTGGAAGGTGACGCTAACGCAGATGCAGCTAACGCGCGGCGTATTCTCACCAAGATTAACCGACATAtt                                                                                                                                                                                                                                                               >  1:103923/1‑301 (MQ=255)`  
                                            `ggCCGCACGAACCCGCGCTGCTAACAAAGCCCGAAAGGAAGCTGAGTTGGCTGCTGCAACCGCTTAGCAATAACTAGCATAACCCCTTGGGGCCTCTAAACGGGTCTTGAGGGGTTTTTTGCTGAAAGGAGGAACTATATGCGCTCATGCGATATGAACGTTGAGACTGCCGCTGAGTTATCAGCTGTGAACGACATTCTGGCGTCTATCGGTGAACCTCCGGTATCAATGCTGGAAGGTGACGCTAACGCAGATGCAGCGAACGCTCGGCGTATTCTCAACAAGATTAACCGACAGATTc                                                                                                                                                                                                                                                              <  1:271223/301‑1 (MQ=255)`  
                                             `gCCGCACGCACCCGCGCTGCTAACAAAGCCCGAAAGGAAGCTGAGTTGGCTGCTGCCACCGCTGAGCAATAACTAGCATAACCCCTTGGGGCCTCTAAACGGGTCTTGAGGGGTTTTTTGCTGAAAGGAGGAACTATATGCGCTCATGCGATATGACCGTTGAGACTGCCGCTGCGTTATCAGCTGTTAACGACATTCTGGCGTCTATCGGTGAACCTCCGGTATCAACGCTGGAAGGTGACGCTAACGCAGATGCAGCGAACGCTAGGCGTATTCTCAACAAGATTAACCGACATATTCa                                                                                                                                                                                                                                                             >  1:146023/1‑301 (MQ=255)`  
                                              `ccGCACGCACCCGCGCTGCTAACAAAGCCCGAAAGGAAGCTGAGTTGGCTGCTGCCACCGCTGAGCAATAACTAGCATAACCCCTTGGGGCCTCTAAACGGGTCTTGAGGGGTTTTTTGCTGAAAGGAGGCACTATATGCGCTCATACGATATGAACGTTGCGACTGCCGCTGAGTTATCAGCTGTGAACGACATTCTGGCGTCTATCGGTGAACCTCCGGTATCAACGCTGGCAGGTGACGCTAAACCAGACGCAGCGAACGCTCGGCGTAGTCTCAACAAAATTAACCAACAGATTCaa                                                                                                                                                                                                                                                            >  1:208217/1‑301 (MQ=255)`  
                                                `gcacgcacCCGCGCTGCTAACAAAGCCCGAAAGGAAGCTGAGTTGGCTGCTGCCACCGCTGAGCAATAACTAGCATAACCCCTTGGGGCCTCTAAACGGTTCTTGAGGGGTTTTTTGCTGAAAGGAGGAACTATATGCGCTCATACGATATGAACGTTGAGACTGCCGCTGAGTAATCAGCTGGAAACGACATTCTGGCGTCTATAGGTGAACCTCCGGTATCAACGCTGGAAGGTGACTATAACGCAAATGTAGCTCACGCTCGTCCTATTCCTAAACAGATTAACTGGAAGATTTaaat                                                                                                                                                                                                                                                          >  1:410087/1‑299 (MQ=255)`  
                                                `gcacgcacCCGCGCTGCTAACAAAGCCCGAAAGGAAGCTGAGTTGGCTGCTGCCACCGCTGAGCAATAACTAGCATAACCCCTTGGGGCCTCTAAACGGGTCTTGAGGGGTTTTTTGCTGAAAGGAGGAACTATATGCGCTCATACGATATGAACGTTGAGACTGCCGCTGAGTTATCAGCTGTGAACGACATTCTGGCGTCTATCGGTGAACCTCCGGTATCAACGCTGGAAGGTGACGCTAACGCAGATGTAGCGAACGCTCGGCGTATTCTCAACAAGATTAACCGACAGATTCAAtc                                                                                                                                                                                                                                                          <  1:198086/301‑1 (MQ=255)`  
                                                 `cacgcacCCGCGCTGCTAACAAAGCCCGAAAGGAAGCTGAGTTGGCTGCTGCCACCGATGAGCAATAACTAGCATAACCCCTTGGGGCCTCTAAACGGGTCTTGAGGGGTTTTTTGCTGAAAGGAGGAACTATATGCGCTCATGCGATATGAACGTTGAGACTGCCGCTGAGTTATCAGCTATGAACGACATTCTGGCGTCTATCGGTGAACCTCCGGTATCAACGCTGGAAGGTGACGCTAACGCAGATGCAGCGAACGCTCGGCGTATTCTCAACAAGATTAACCGACATATTCAAtct                                                                                                                                                                                                                                                         <  1:131117/301‑1 (MQ=255)`  
                                                     `cacCCGCGCTGCTAACAAAGCCCGAAAGGAAGCTTAGTTGGCTGCTGCCACCGCTGAGCAATAACTAGCATAACCCCTTGGGGCCTCTAAACGGGTCTTGAGGGGTTTTTTGCTGAAAGGAGGAACTATATGCGCTCATACGATATGAACGTTGAGACTGCCGCTGAGTTATCAGCTGTGGACGACATTCTGGCGGCTATCGGTTAACCTCCCGTATCAACGCTTGAAGGTGACGCTAACTCACATGTACCCAACACTCCGCGTATACTCCACAAGATTAACCGACCGATTCAAtcccctg                                                                                                                                                                                                                                                     >  1:89492/1‑296 (MQ=255)`  
                                                     `cacCCGCGCTGCTAACAAAGCCCGAAAGGAAGCTGAGTTGGCTGCTGCCACCGCTGAGCAATAACTAGCATAACCCCTTGGGGCCTCTAAACGGGTCTTGAGGGGTTTTTTGCTGAAAGGAGGAACTATATGCGCTCATACGATATGAACGTTGAGACTGCCGCTGAGTTATCAGCTGTGAACGACATTCTGGCGTCTATCGGTGAACCTCCGGTATCAACGCTGGAAGGTGACGATAACGCAGATGTAGCGAACGCCCGGCGCATTCTCAACAAGATTAACCGACAGATTCAAtatctgg                                                                                                                                                                                                                                                     >  1:95625/1‑298 (MQ=255)`  
                                                             `cttctAACAAAGCCCGAAAGGAAGCTGAGTTGGCTGCTGCCACCGCTGAGCAATAACTAGCATAACCCCTTGGGGCCTCTAAACGGGTCTTGAGGGGTTTTTTGCTGAAAGGAGGAACTATATGCGCTCATACGATATGAACGTTGAGACTGCCGCTGAGTTATCAGCTGTGAACGACATTCTGGCGTCTATCGGTGAACCTCCGGTATCAACGCTGGAAGGTGACGCTAACGCAGATGTAGCGAACGCTCGGCGTATTCTCAACAAGATTAACCGACAGATTCAATCTCGTGGATGGACg                                                                                                                                                                                                                                             <  1:67843/298‑1 (MQ=255)`  
                                                             `ctgctAACAAAGCCCGAAAGGAAGCTGAGTTGGCTGCTGCCACCGCTGAGCAATAACTAGCATAACCCCTTGGGGCCTCTAAACGGGTCTTGAGGGGTTTTTTGCTGAAAGGAGGAACTATATGCGCTCATACGATATGAACGTTGATAATGCCGCTGAGTTATCAGCTGTGAACGAAATTCTGGCGTCTATCGGTGAACCTCCGGTATCAACGCTGGAAGGTAAAGCTAAAGCAGATGTAGCCAACGCTCGGCGTATTCTCAACAAGATTAACCGACCGATTCAATCTCGTGGCTGGACg                                                                                                                                                                                                                                             >  1:120843/1‑301 (MQ=255)`  
                                                                     `aaaGCCCGAAAGGAAGCTGAGTTGGCTGCTGCCACCGCTGAGCAATAACTAGCATAACCCCTTGGGGCCTCTAAACGGGTCTTGAGGGGTTTTTTGCTGAAAGGAGGAACTATATGCGCTCATACGATATGAACGTTGAGACTGCCGCTGAGTTATCAGCTGTGAACGACATTCTGGCGTCTATCGGTGAACCTCCGGTATCAACGCTGGAAGGTGACGCTAACGCAGATGTAGCGAACGCTCGGCGTATTCTCAACAAGATTAACCGACAGATTCAATCTCGTGGATGGACGTTCAACAt                                                                                                                                                                                                                                     <  1:478703/301‑1 (MQ=255)`  
                                                                        `gCCCGAAAGGAAGCTGAGTTGGCTGCTGCCACCGCTGAGCAATAACTAGCATAACCCCTTGGGGCCTCTAACCGGGTCCTGAGGGGTTTTTTGCTGAAACGAGGAACTATATGCGCTCATACGATATGAACGTTGAGAACGCCGCTGAGTTATATGCTGTATACGACATTCAGGCCACTCGCGCTGAACCTCCGGTCCTAAACCTGGAAGGTGACGATAACGCAGCTGTCGCAAACTCTCCGCGCATTCCCAACAATATTAGCCTCTAAATTCAATCTCCTCGCTGTACATTCAACCTTGa                                                                                                                                                                                                                                  >  1:419256/1‑301 (MQ=255)`  
                                                                        `gCCCGAAAGGAAGCTGAGTTGGCTGCTGCCACCGCTGAGCAATAACTAGCATAACCCCTTGGGGCCTCTAAACGGGTCTTGAGGGGTTTTTTGCTGAAAGGAGGAACTATATGCGCTCATACGATATGAACGTTGAGACTGCCGATGAGTTATCAGCTGTGCACGACATCCTGGCGTCTATCGGTGAACCTTCGGTATCAACGCTGTAAGGTGACGCTAACGCAGCTGTAGCGAACGCTCGGCGTATTATCAACAAGCTTAACCGACAGATTCAATCTCGTGACTTGACGTTAAACATTGa                                                                                                                                                                                                                                  >  1:56187/1‑301 (MQ=255)`  
                                                                             `aaaGGAAGCTGAGTTGGCTGCTGCCACCGCTGAGCAATAACTAGCATAACCCCTTTGGGCCTCTAAACGGGTCTTGATGGGTTTTTTGCTGAAAGGAGGAACTATATGCGCTCATACGATATGAACGTTGAGACTGCCGCTGAGTTATCACCTGTGAACGACATTCTGGCGTCTATCGGTTAACCTCCGGTATCAACGCTGGAAGTTGACGCTAACGCAGATCTCGCGAACGTCCGGCGTATTCTCAACAAGATTAACCGAAAGATTCACTCTCGTGGCTGGACGGTCAACATTGAggctt                                                                                                                                                                                                                             >  1:259860/1‑298 (MQ=255)`  
                                                                             `aaaGGAAGCTGAGTTGGCTGCTGCCACCGCTGAGCAATAACTAGCATAACCCCTTGGGGCCTCTAAACGGGTCTTGAGGGGTTTTTTGCTGAAAGGAGGAACTATATGCGCTCATACGATATGCACGTTGAGACTGCCGCTCAGTTATCAGCTGTGTACGACCTTCTGGCGTCTATCGGTGAACCTCCGGTATCAACGCTGGAAGGTGACGCTAACGCAAATGTATCTAACGCCCGCAGTATTCTCAACACGATTAACCGACAGTTTCATTCTCCTTCCTTGACGTGCACACTTGAGGAAg                                                                                                                                                                                                                             >  1:251148/1‑301 (MQ=255)`  
                                                                             `aaaGGAAGCTGAGTTGGCTGCTGCCACCGCTGAGCAATAACTAGCATAACCCCTTGGGGCCTCTAAACGGGTCTTGAGGGGTTTTTTGCTGAAAGGAGGAACTATATGCGCTCATACGATATGAACGTTGAGACTGCCGCTGAGTTATCAGCTGTGAACGACGTTCTGGCGTCTATCGGTGAACCTCCGGTATCAACGCTGGAAGGTGACGCTAACGCAGATGTAGCGAACGCTCGGCGTAATCTCAACAAGATTAACCGACAGATTCAATCTCGTGGATGGACGTTCAACATTTAGGacg                                                                                                                                                                                                                             >  1:469005/1‑299 (MQ=255)`  
                                                                                      `tGAGTTGGCTGCTGCCACCGCTGAGCAATAACTAGCATAACCCCTTGGGGCCTCTAAACGGGTCTTGAGGGGTTTTTTGCTGAAAGGAGGAACTATATGCGCTCATACGATATGAACGTTGAGACTGCCGCTGAGTTATCAGCTGTGAACGACATTCTGGCGTCTATCGGTGAACCTCCGGTATCAACGCTGGAAGGTGACGCTAACGCAGATGTAGCGAACGCTCGGCGTATTCTCAACAAGATTAACCGACAGATTCAATCTCGTGGATGGACGTTCAACATTGAGGAAGGCATAACGc                                                                                                                                                                                                                    <  1:211035/301‑1 (MQ=255)`  
                                                                                       `gAGTTGGCTGCTGCCACCGCTGAGCAATAACTAGCATAACCCCTTGGGGCCTCTAAACGGGTCTTGAGGGGTTTTTTGCTTAAAGGAGGAACTATATGCGCTCATACGATATGAACGTTGAGACTGCCGCTGAGTTATCAGCTGTGAACGACATTCTGGCGTCTATCGGTGAACCTCCGGTATCAACGCTGGAAGGTGACGCTAACGCAGATGTAGCGAACGCTCGGCGTATTCTCAACAAGATTAACCGACAGATTCAATCTCGTTGATGGACGTTCAACATTGAGAAAGGCATAACGct                                                                                                                                                                                                                   >  1:29613/1‑301 (MQ=255)`  
                                                                                       `gAGTTGGCTGCTGCCACCGCTGAGCAATAACTAGCATAACCCCTTGGGGCCTCTAAACGGGTCTTGAGGGGTTTTTTGCTGAAAGGAGGAACTATATGCGCTCATACGATATGAACGTTGAGACTGCCGCTGAGTTATCAGATGTTAACGACATTCTGGCGTCTATCGGTGAACCTACGGTATCAACGCTGGAAGGTGACGCTAACGCAGCTGTAGCGAACGCCCGGCGTATTCTCAACAAGATTAACCGACAGATTCAATCTCGTGGATGGACGTTCAACATTGAGGAAGGCATAAcccc                                                                                                                                                                                                                   >  1:500933/1‑298 (MQ=255)`  
                                                                                            `ggCTGCTGCCACCGCTGAGCAATAACTAGCATAACCCCTTGGGGCCTCTAAACGGGTCTTGAGGGGTTTTTTGCTGAAAGGAGGAACTATATGCGCTCATACGATATGAACGTTGAGACTGCCGCTGAGTTATCAGCTGTGAACGACATTCTGGCGTCTATCGGTGAACCTCCGGTATCAACGCTGGACGGTGACACTAACGCAGATGTAGCGAACGCTCGGCGTATTCTCAACAAGATTAACCGACAGATTCAATCTCGTGGATGGACGTTCAACATTGAggctgtctcttatacacatc                                                                                                                                                                                                              >  1:397735/1‑283 (MQ=255)`  
                                                                                            `ggCTGCTGACACCGCTTAGCAATAACTAGCATAACCCCTTGGTGCCTCTAAACGGGTCTTGAGGGGTTTTTTGCTGAAAGGAGGAACTATATGCGCTCATACGATATTAACGTTGAGACTGCCGCTGAGTTATCAGCTGTGAACGACATTCTGGCGTCTATCGGTGAACCTCCTGTATCAACGCTGGAAGGTGACGCTAACGCAGATGCAGCGAACGCTCGGCGTATTCTCAACAAGATTAACCGACATATTCAATCTCGTGGATGGACGTTCAACATTGAGGAAGGCATAACGCtactac                                                                                                                                                                                                              <  1:218567/301‑1 (MQ=255)`  
                                                                                             `agttgtataagagacagtcAATAATTAGCATAACCCCTTGGGGCCTCTAAACGGGTCTTGAGGGGTTTTTTGCTGAAAGGAGGAACTATATGCGCTCATACGATATGAACGTTGAGACTGCCGCTGAGTTATCAGCTGTGAACGACATTCTGGCGTCTATCGGTGAACCTCCGGTATCAACGCTGGAAGGTGACGCTAACGCAGATGTAGCGAACGCTCGGCGTATTCTCAACAAGATTAACCGACAGATTCAATCTCGTGGATGGACGTTCAACATTGAGGAAGGCATAACGCTACTAcc                                                                                                                                                                                                             <  1:226359/283‑1 (MQ=255)`  
                                                                                              `ctgctgCCACCGCGGATCAATAACCAGCCTAACCCCTTTGGGCCTCTAAACGGGTCTTGAGGGGTTTTTTGCTGAAAGGAGGAACTATATGCGCTCATGCGATATGAACGTTGAGACTGCCGCTGAGTTATCAGCTGTGAACGACATTCTGGCGTCTATCGGTGAACCTCCGGTATCAACGCTGGAAGGTGACGCTAACGCAGATGCAGCGAACGCTCGGCGTATTCTCAACAAGATTAACCGACATATTCAATCTCGTGGATGGACGTTCAACATTGAGGAAGGCATAACGCTACTACCt                                                                                                                                                                                                            <  1:126327/301‑1 (MQ=255)`  
                                                                                                `gctacCACCGCTGAGCAATAACTAGCATAACCCCTTGGGGCCTCTAAACGGGTCTTGAGGGGTTTTTTGCTGAAAGGAGGAACTATATGCGCTCATGCGATATGAAAGTTGAGACTGCCGCTGAGTTATCAGCTGTGAACGACATTCTGGCGTCTAACGGTGAACCTTCGGTATCAACGCTGGAAGGTGACGCTAACGCAGATGCAGCGAACGCTCGGCGTATTCTCAACACGATTAACCGACATATTCAATCTCGTGGATGGACGTTCAACAtctgtctcttatacacatctccgagccc                                                                                                                                                                                                          >  1:483340/1‑274 (MQ=255)`  
                                                                                                 `ctgcCACCGCTGAGCAATAACTAGCATAACCCCTTGGGGCCTCTAAACGGGTCTTGAGGGGTTTTTTGCTGAAAGGAGGAACTATATGCGCTCATACGATATGAACGTTGAGACTGCCGCTGCGTTATCAGCTGTGAACGACATTCTGGCGTCTATCGGTGAACCTCCGGTATCAACGCTGGAAGGTGACGCTAACTCAGATGTAGCGAACGCTCGTCGTATTCTTAACAAGATTAACCGACAGATTCAATCTCGTGGATGGACGTTCAACATTGATCTAGCCATATCCCTACTACCTCAt                                                                                                                                                                                                         >  1:254751/1‑301 (MQ=255)`  
                                                                                                  `taagagacaggAGCAATAACTAGCATAACCCCTTGGGGCCTCTAAACGGGTCTTGAGGGGTTTTTTGCTGAAAGGAGGAACTATATGCGCTCATACGATATGAACGTTTAGACTGCCGCTGAGTTATCAGCTGTGAACGACATTCTGGCGTCTATCGGTGAACCTCCGGTATCAACGCTGGAAGGTGACGCTAACGCAGATGTAGCGAACGCTCGGCGTATTCTCAACAAGATTAACCGACAGATTCAATCTCGTGGATGGACGTTCAACATTGAGGAAGGCATAACGCTACTACCTGATg                                                                                                                                                                                                        <  1:193355/291‑1 (MQ=255)`  
                                                                                                       `ccGCTGAGCAATAACTAGCATAACCCCTTGGGGCCTCTAAACGGGTCTTGAGGGGTTTTTTGCTGAAAGGAGGAACTATATGCGCTCATACGATATGAACGTTGAGACTGCCGCTGAGTAATCAGCTGTGAACGACATTCTGGCGTCTATAGGTGAACCTCCGGTATCAACTCTGGAAGGTGACACTAACGCAGATGTAGCGAACGCTCGGCCTATTCTCAACATGATTCACCGACAAATTCAATCTCATGGATTGCTGTTCAACCTTTAGGAAGTCATAAcccacatactttagttttac                                                                                                                                                                                                   >  1:382788/1‑282 (MQ=255)`  
                                                                                                         `gCTGAGCAATAACTAGCATAACCCCTTGGGGCCTCTAAACGGGTCTTGAGGGGTTTTTTGCTGAAATGAGGAACTATATGCGCTCATACGATATGAACGTTGAGACTGCCGCTGAGTTATCAGCTGTGAACGACATTCTGGCGTCTATCGGTGAACCTCCGGTATCAACGCTGGAAGGTGACGCTAACGCAGATGTAGCGAACGCTCGGCGTATTCTCAACAAGATTAACCGACAGATTCAATCTCGTGGATGGACGTTCAACATTGAGGAAGGCATAACGCTACTACCTGATGTTTACTc                                                                                                                                                                                                 <  1:427551/301‑1 (MQ=255)`  
                                                                                                          `tcggagatgtgtataagagacagcTTGGGGCCTCTAAACGGGTCTTGAGGGGTTTTTTGCTGAAAGGAGGAACTATATGCGCTCATACGATATGAACGTTGAGACTGCCGCTGAGTTATCAGCTGTGAACGACATTCTGGCGTCTATCGGTGAACCTCCGGTATCAACGCTGGAAGGTGACGCTAACGCAGATGTAGCGAACGCTCGGCGTATTCTCAACAAGATTAACCGACAGATTCAATCTCGTGGATGGACGTTCAACATTGAGGAAGGCATAACGATACTACCTGATGTTTACTcc                                                                                                                                                                                                <  1:2013/278‑1 (MQ=255)`  
                                                                                                          `gtataagagacagaGCATAACCCCTTGGGGCCTCTAAACGGGTCTTGAGGGGTTTTTTGCTGAAAGGAGGAACTATATGCGCTCATGCGATATGAACGTTGAGACTGCCGCTGAGTTATCAGCTGTGAACGACATTCTGGCGTCTATCGGTGAACCTCCGGTATCAACGCTGGAAGGTGACGCTAACGCAGATGCAGCGAACGCTCGGCGTATTCTCAACAAGATTAACCGACATATTCAATCTAGTGGATGGACGTTCAACATTGAGGAAGGCATAACGCTACTACCTGATGTTTACTcc                                                                                                                                                                                                <  1:164536/288‑1 (MQ=255)`  
                                                                                                            `gAGCAATAACTAGCATAACCCCTTGGGGCCTCTAAACGGGTCTTGAGGGGTTTTTTGCTGAAAGTAGTAACTCTATGCGCTCATACGATATGAACGTTGAGACTGCCGCTGAGTTCTCAGCTGTGAACGACATTCTTGCGTCTATCTGTGAACCTCCGGTATCCACTCTGGTAGGTGACGCTAACGCAGATGTACCTACCTCTCGGCGTATTCTCAACACTATTCACCGACAGATTCAATCTCGTTGATTGACTTTCACCATTTATGAAGGCATACCTCTCCTACCTGATGTTTACTccta                                                                                                                                                                                              >  1:161687/1‑299 (MQ=255)`  
                                                                                                                     `cTAGCATACCCCCTTGGGGCCTCTAAACGGGTCTTGAGGGGTTTTTTGCTGAAAGGAGGAACTATATCCGCTCATACGATCTGCACGTTGAGACTGCCGCTGAGTTTTCAGCTGTGAACGACCTTCTGGCGTCTATCGGTGAACATCCGGTATCAACGCTGGAAGGTGACGCTAAAGCATATGTAACGAACGCTCGTCGTATTCTCAACAAGATTAACCGACAGCTTCAATCTCGTGGCTGGACGTTCAACATTTATGAAGGCATAACACCACTCCCTTATGTTTCCTCCAACCTACTTGt                                                                                                                                                                                     >  1:369602/1‑301 (MQ=255)`  
                                                                                                                       `aGCCTAACCCCTTGGGGCCTCTAAACGGGGCTTGAGGGGTTTTTTGCTGAAAGGAGGAACTATAGTGTCTCATACGATATGAACGTTGAGACTGCCGCTGAGTTATCAGCTGTGAACGACATTCTGGCGTCTATCGGTGAACCTCCGGTATCAACGCTGGAAGGTTACGCTAACGAAGATGTAGCGAACGCTCGGCGTATTCTCAACAAGATTAACCGACAGATTCAATCTCGTGGATGGACGTTCAACATTGAGGAAGGCATAACGCTACTACCTGATGTTTACTCCAACCTGATTGtat                                                                                                                                                                                   <  1:319932/301‑1 (MQ=255)`  
                                                                                                                         `cATAACCCCTTGGGGCCTCTAAACGGGTCTTGAGGGGTTGTTTGCTGAAAGGAGGAACTATATGCGCTCATACGATATGAACGTTGAGACTGCCGCTGAGTTATCAGCTGTGAACGACATTCTGGCGGCTATCGGTGAACCTCCGGTATCAACGCTGGAAGGTGACGCTAACGCAGATGTAGCGAACGCTCGGCGTATTCTCAACAAGATTAACCGACAGATTCAATCTCGTGGATGGACGTTCAACATTGAGGAAGGCATAACGCTACTACCTGATGTTTACTCCAACCTGATTGTATAc                                                                                                                                                                                 <  1:230131/301‑1 (MQ=255)`  
                                                                                                                         `acagaCCCCTTGGGGCCTCTAAACGGGTCTTGAGGGGTTTTTTGCTGAAAGGAGGAACTATATGCGCTCATACGATATGAACGTTGAGACTGCCGCTGAGTTATCAGCTGTGAACGACATTCTGGCGTCTATCGGTGAACCTCCGGTATCAACGCTGGAAGGTGACGCTAACGCAGATGTAGCGAACGCTCGGCGTATTCTCAACAAGATTAACCGACGGATTCAATCTCGTGGATGGACGTTCAACATTGAGGAAGGCATAACGCTACTACCTGATGTTTACTCCAACCTGATTGTATAc                                                                                                                                                                                 <  1:443106/297‑1 (MQ=255)`  
                                                                                                                           `tAACCCCTTGGGGCCTCTAAACGGGTCTTGAGTGGTTTTTTGCTGAAAGGAGGAACTATATGCGCTCATGCGATATGAACGTTGAGACTGCCGCTGAGTTATCAGCTGTGAACGACATTCTGGCGTCTATCGGTGAGCCTCCGGTATCAACGCTGGAAGGTGACGCTAACGCAGATGCAGCGAACGCTCGGCGTATTCTCAACAAGATTAACCGACATATTCAATCTCGTGGATGGACGTTCAACATTGAGGAAGGCATAACGCTACTACCTGATGTTTACTCCAACCTGATTGTATACAg                                                                                                                                                                               <  1:174067/301‑1 (MQ=255)`  
                                                                                                                           `tAACCCCTTGGGGCCTCTAAACGGGTCTTGAGGGGTGTTTTGCTGAAAGGAGGAACTATATGCTCTCATGCGATATGAACGTTGAGACTGCCGCTGAGTTATCAGCTGTGAACGACATTCTGGCGTCTATCGGTGAACCTCCGGTATCAACGCTGGAAGGTGACGCTAACGCAGATGCAGCGAACGCTCGGCGTATTCTCAACAAGATTAACCGACATATTCAATCTCGTGGATGGACGTTCAACATTGAGGAAGGCATAACGCTACTACCTGATGTTTACTCCAACCTGATTGTATACAg                                                                                                                                                                               <  1:309662/301‑1 (MQ=255)`  
                                                                                                                              `ccccTTGGGGCCTCTAAACTGGTCTTGAGTGGTTTTTTGCTGAAAGGAGGAACTATATGCGCTCATACGATATGAACGTTGAGACTGCCGCTGAGTTCTCAGCTGTGAACGACATTCTGGCGTCTATCGGTGAACCTCCGGTATCAACGCTGGAAGGTGACGCTAACGCAGATGTATCGAACGCTAGGCGTATTCTCAACAAGATTAACAGACAGATTAAATCTCGTGGATGGACGTTCAACTTTGAGGAAGGCATAACGCTACTACCTGATGTTTACTCCAACCTGATTGTATACAGTgt                                                                                                                                                                            >  1:149545/1‑300 (MQ=255)`  
                                                                                                                              `ccccTAGGGGCCTATAAACGGGTCTGGAGGGGTTTTTTGCTGAAAGGAAGAACAAGATGCGCTCATACGATATGGACGTTGAGACTGCCGCTGAGTTATCAGCTGTGAACGACATTCTGGCGTCTAACGGTGAACCTCCGGTATCAACGCTGGAAGGTGACGCTAACGCAGATGTAGCGAACGCTGGGCGTATTCTCAACAAGATTAACCGACAGATTCAATCTCGTGGATGGACGTTCAACATTGAGGAAGGCATAACGCTACTACCTGATGTTTACTCCAACCTGATTGTATACAGTga                                                                                                                                                                            <  1:403405/301‑1 (MQ=255)`  
                                                                                                                               `cccTTGGGGCCTCTAAACGGGTCTTGAGGGGTTTTTTGCTGAAAGGAGGAACTATATGCGCTCATACGATATGAACGTTGAGACTGCCGATGAGTTATCAGCTGTGAACGACATTCTGGCGTCTATCGGTGAACCTCCGGTATCAACGCTGGAAGGTGACGCTAACGCAGATGTAGCGAACGCTCGGCGTATTCTCAACAAGATTAACCGACAGATTCAATCTCGTGGATGGACGTTCAACATTGAGGAAGGCATAACGCTACTACCTGATGTTTACTCCAACCTGATTGtatctgtctct                                                                                                                                                                           >  1:423783/1‑293 (MQ=255)`  
                                                                                                                                `ccTTGGGGCCTCTAAACGGGTCTTGAGTGGTTTTTTGCTGAAAGGAGGAACTATATGCGCTCATACGATATGAACGTTGAGACTGCCGCTGAGTTATCAGCTGTGAACGACATTATGGCGTCTATCGGTGAACCTCCGGTATCAACGCTAGAAGGTGACGCTAACGCAGATGTAGCGAACCCTCGGCGTATTATCAACAAGATTAACCAACCGATTCAATCTCTTGGATGGACGTTCAACATTGAGGAAGGCATAAAGCTCCTACCTGATGTTTACTCAAACCTCCTTCTATCCAGTgacg                                                                                                                                                                          >  1:109167/1‑301 (MQ=255)`  
                                                                                                                                  `ttGGGGCCTCTAAACGGGTCTTGAGGGGTTTTTTGCTGAAAGGAGGAACTATATGCGCTCATACGATATGAACGTGGAGACTGCCGCTGAGTTATCAGCTGTGAACGACATTCTGGCGTCTATCGGTGAACCTCCGGTATCAACGCTGGAAGGTGACGCTAACGCAGATGTAGCGAACGCTCGGCGTATTCTCAACAAGATTAACCGACAGATTCAATCTCGTGGATGGACGTTCAACATTGAGGAAGGCATAACGCTACTACCTGATGTTTACTCCAACCTGATTGTATACAGTgacgac                                                                                                                                                                        <  1:117379/301‑1 (MQ=255)`  
                                                                                                                                   `tGGGGCATCTAAACGGGTCTTGAGGGGTTTTTTGCTGAAAGGAGGAACTATATGCGCTCATACGATATGAACGTTGAGACTGCCGCTGAGTTATCAGCTGTGAACGACATTCTGGCGTCTATCGGTGAACCTCCGGTATCAACGCTGGAAGGTGACGCTAACGCAGATGTAGCGAACGCTCGGCGTATTCTCAACAAGATTAACCGACAGATTCAATCTCGTGGATGGACGTTCAACATTGAGGAAGGCATAACGCTACTACCTGATGTTTACTCCAACCTGATTGTATACAGTGACGACt                                                                                                                                                                       <  1:181984/301‑1 (MQ=255)`  
                                                                                                                                    `acagCCTCTAAACGGGTCTTGAGGGGTTTTTTGCTGAAAGGAGGAACTATATGCGCTCATACGATATGAACGTTGAGACTGCCGCTGAGTTATCAGCTGTGAACGACATTCTGGCGTCTATCGGTGAACCTCCGGTATCAACGCTGGAAGGTGACGCTAACGCAGATGTAGCGAACGCTCGGCGTATTCTCAACAAGATTAACCGATAGATTCAATCTCGTGGATGGACGTTCAACATTGAGGAAGGCATAACGCTACTACCTGATGTTTACTCCAACCTGATTGTATACAGTGACGACTa                                                                                                                                                                      <  1:463637/298‑1 (MQ=255)`  
                                                                                                                                       `gCCTCTAAACGGGTCTTGAGGGGTTTTTTGCTGAAAGGAGGAACTATATGCGCTCATGCGATATGAACGTTGAGACTGCCGCTGAGTTATCAGCTGTGAACGACATTCTGGCGTCTATCGTTTAACCTCCGGTATCAACGCTGGACGGTGACGCTAACGCAGATGCAGCGAACGCTCGGCGTATTCTCAACAAGATTAACCGACATATTCAATCTCGTGGATGGACGTTCAACATTGAGGAAGGCATAACGCTACTACCTGATGTTTACTCCAACCTGATTGTATAcctgtctcttatacc                                                                                                                                                                   >  1:24619/1‑287 (MQ=255)`  
                                                                                                                                       `gCCTCTAAACGGGTCTTGAGGGGTTTTTTGCTGAAAGGAGGAACTATATGCGCTCATACGATATGAACGTTGAGACTGCCGCTGAGTTATCAGCTGTGAACGACATTCTGGCGTCTATCGGTGAACCTCCGGTATCAACGCTGGAAGGTGACGCTAACGCAGATGTAGCGAACGCTCGGCGTATTCTCAACAAGATTAACCGACAGATTCAATCTCGTTGATGGACGTTCAACATTGAGGAAGGCATAACGCTACTACCTGATGTTTACTCCAACCTGATTGTATACAGTGACGACTAttt                                                                                                                                                                   <  1:344431/301‑1 (MQ=255)`  
                                                                                                                                          `gctAAAAGGGTTGTGAGGGTTATTTTGCTGAAAGGAGGAACTATATGCGCTCATTCGATATGAACGTTGAGACTGCCGCTGAGTTATCAGCTGTGAACGACATTCTGGCGTCTATCGGTGAACCTCCGGGATCAACGCTGGAAGGTGACGCTAAAGCAGATGCAGCGAACGCTCGGCGTATTCTCAACAAGAGTAACCGACATATTCAATCTCGTGGATGGACGTTCAACATTGAGGAAGGCATAACGCTACTACCTGATGTTTACTCCAACCTGATTGTATACAGTGACGACTATTTATc                                                                                                                                                                <  1:73149/300‑1 (MQ=255)`  
                                                                                                                                           `ctAAACGGGTCTTGAGCGGTTTTTTGCTGAAAGGAGGAACTATATGCGCTCATACGATATGAACGTTGAGACTGCCGCTGAGTTATCAGCTGTGAACGACATTCTGGCGTCTATCGGTGAACCTCCGGTATCAACGCTGGAAGGTGACGCTAACGCAGATGTAGCGAACGCCCGGCGTATTCTCAACAAGATTAACCGACAGATTCAATCTCGTGGATGGACGTTCAACATTGAGGAAGGCATAACGCTACTACCTGATGTTTACTCCAACCTGATTGTATACAGTGACGACTATTTATcc                                                                                                                                                               >  1:275606/1‑301 (MQ=255)`  
                                                                                                                                             `aaaCGGGTCTTGAGGGGTTTTTTGCTGAAAGGAGGAACTATATGCGCTCATACGATATGAACGTTGAGACTGCCGCTGAGTTATCAGCTGTGAACGACATTCTGGCGTCTATCGGTGAACCTCCGGTATCAACGCTGGAAGGTGACGCTAACGCAGATGTAGCGAACGCTCGGCGTATTCTCAACAAGATTAACCGACAGATTCAATCTCGTGGATGGACGTTCAACATTGAGGAAGGCATAACGCTACTACCTGATGTTTCCTCCAACCTGATTGTATACAGTgactacctgtctcttat                                                                                                                                                             >  1:374108/1‑290 (MQ=255)`  
                                                                                                                                                `cGGGTCTTGAGGGGTTTTTTGCTGAAAGGAGGAACTATAGGCGCTCATACGATATGAACGTTGAGACTTCCGCTGGGTTATCAGCTGTGAACGACATTCTGGCGTCTATCGGTGAACCTCCGGTATAAACGCTGAAAGGTGACGGTAACGCACATGTAGCGAACGCTCGGCGTATTCTCAACAAGATTAACCGACAGTTTCAATCTCGTGGTTGATCGTTCAACATTGGGGAAGGCATAACGCTACTACCTTCTTTTTTCTCCACCCTATTCTTATACCTTACCCACTATTTTTCCCTAAt                                                                                                                                                          >  1:18566/1‑301 (MQ=255)`  
                                                                                                                                                  `ggTCTTGAGGGGTTTTTTGCTGAAAGGAGGAACTATATGCGCTCATACGATATGAACGTTGAGACTGCCGCTGAGTTATCAGCTGTGAACGACATTCTGGCGTCTATCGGTGAACCTCCGGTATCAACGCTGGAAGGTGACGCTAACGCAGATGTAGCGAACGCTCGGCGTATTCTCAACAAGATTAACCGACAGATTCAATCTCGTGGATGGACGTTCAACATTTAGGAAGGCATAACGCTACTACCTTATGTTTACTCCAACCTGATTGTATACAGTGACGACTATTTATCCCtctgtc                                                                                                                                                        >  1:405778/1‑296 (MQ=255)`  
                                                                                                                                                      `ttGAGGGCTTTTTTTCTGAAAGGAGGAACTATATGCGCTCATACGATATGAACGTTGAGACTGACTCTGAGTTAACAGCTGTGAATGACAGTCTGGCGTCTATCGGTTAACCTCCGGTATCAACGCTGGAAGGTGACGCTAACGCAGATGTAGCGAACGCTCGGCGTATTCTCAACAAGATTAACCGACAGATTCAATCTCGTGGATTGACGTTCAACATTGAGGAAGGCATAACGCTACTACCTGATGTTTACTCCAACCTGATTGTATACAGTGACGACTATTTATCCCTAATGTCTAc                                                                                                                                                    <  1:267701/301‑1 (MQ=255)`  
                                                                                                                                                         `gggggTTTTTTGCTGAAATGAGGAACTATATGCGCTCATGCGATATGAACGTTGAGACTGCCGCTGAGTTATCAGCTGTGAACGACATTCTGGCGTCTATCGGTGAACCTCCGGTATCAACGCTGGAAGGTGACGCTAACGCAGATGCAGCGAACGCTCGGCGTATTCTCAACAAGATTAACCGACATATTCAATCTCGTGGATGGACGTTCAACATTGAGGAAGGCATAACGCTACTACCTGATGTTTACTCCAACCTGATTGTATACAGTGACGACTATTTATCCCTAATGTCTACTTc                                                                                                                                                 <  1:28593/300‑1 (MQ=255)`  
                                                                                                                                                         `atgtgtataagagacagaggaggAACTATATGCGCTCATGCGATATGAACGTTGAGACTGCCGCTGAGTTATCAGCTGTGAACGACATTCTGGCGTCTATCGGTGAACCTCCGGTATCAACGCTGGAAGGTGACGCTAACGCAGATGTAGCGAACGCTCGGCGTATTCTCAACAAGATTAACCGACAGATTCAATCTCGTGGATGGACGTTCAACATTGAGGAAGGCATAACGCTACTACCTGATGTTTACTCCAACCTGATTGTATACAGTGACGACTATTTATCCCTAATGTCTACTTc                                                                                                                                                 <  1:291493/284‑1 (MQ=255)`  
                                                                                                                                                                   `atgtgtataagagacagatatGCGCTCATACGATATGAACGTTGAGACTGCCGCTGAGTTATCAGCTGTGAACGACATTCTGGCGTCTATCGGTGAACCTCCGGTATCAACGCTGGAAGGTGACGCTAACGCAGATGTAGCGAAGGCTCGGCGTATTCTCAACAAGATTAACCGACAGATTCAATCTCGTGGATGGACGTTCAACATTGAGGAAGGCATAACGCTACTACCTGATGTTTACTCCAACCTGATTGTATACAGTGACGACTATTTATCCCTAATGTATACTTCCGGTCAATcc                                                                                                                                       <  1:155560/284‑1 (MQ=255)`  
                                                                                                                                                                           `ggaggaACTATATGCGCTCATACGATATGAACGTTGAGACTGCCGCTGAGTTATCAGCTGTGAACGACATTCTGGCGTCTATCGGTGAACCTCCGGTATCAACGCTGGAAGGTGACGCTAACGCAGATGCAGCGAACGCTCGGCGTATTCTCAACAAGATTAACCGACATATTAAATCTCGTGGATGGACGTTCAACATTGAGGAAGACATAACGCTACTACCTGATGTTTACTCCAACCTGATTGTATACAGTGACGACTATTTATCCCTAATGTCTACTTCCGGTCAATcctgtctctt                                                                                                                               >  1:455367/1‑293 (MQ=255)`  
                                                                                                                                                                            `gaggaACTATATGCGCTCATACGATATGAACGTTGAGACTGCCGCTGAGTTATCAGCTGTGAACGACATTCTGGCGTCTATCGGTGAACCTCCGGTATCAACGCTGGAAGGTGACGCTAACGCAGATGTAGCGAACGCTCGGCGTATTCTCAACAAGATTAACCGACAGATTCAATCTCGTGGATGGACGTTCAACATTGAGGAAGGCATAACGCTACTACCTGATGTTTACTCCAACCTGATTGTATACAGTGACGACTATTTATCCCTAATGTCTACTTCCTGTctcttatacacatct                                                                                                                              >  1:493948/1‑287 (MQ=255)`  
                                                                                                                                                                            `atatgaaaatgagacggcATACTATATGAACGTTGAGAAGGCCGCTGAGTTATCATCTGTGAACGACATGCTGGCGTCTATCGGTGAACCTCCGGTATCAACGCTGGAAGGTGACGCTAACGCAGATGTAGCGAACGCTCGGCGTATTCTCGAAAAGAATAACCGACAGATTAAATCTCGTGGATGGACGTTCAACATTGAGGAAGGCATAACGCTACTACCTGATGTTTACTCCAACCTGATTGTATACAGTGACGACTATTTATCCCTAATGTCTACTTCCGGTCAATCCATCTACGtt                                                                                                                              <  1:142190/284‑1 (MQ=255)`  
                                                                                                                                                                                          `gcgcATACGATATGAACGTTGAGACTGCCGCTGAGTTATCAGCTGTGAACGACATTCTGGCGTCTATCGGTGAACCTTCGGTATCAACGCTGGAAGGTGACGCTAACGCAGATGTAGCGAACGCTCGGCGTATTCTCAACAAGAGTATCCGACAGATTCAATCTCGTGGATTGACGTTCAACATTGAGGAAGGCATAACGCTACTACCTGATGTTTACTCCAACCTGATTGTATACAGTGACGACTATTTATCCCTAATGTCTACTTCCGGTCAATCCATCTACGTTAACCGAGGTGGCTa                                                                                                                <  1:36551/298‑1 (MQ=255)`  
                                                                                                                                                                                                `gcGATATGAACGTTGAGACTGCCGCTGAGTTATCAGCTGTGAACGACAATCAGGCGTCTATCGGTGAACCTCCGGTATCAACGCTGGAAGGTGACGCTAACGCAGATGCAGCAAACGCTCGGCGTATTCTCAACAAGATTAACCGACATATTCAATCTCGTGGATGGACGTTCAACATTGAGGAAGGCATAACGCTACTACCTGATGTTTACTCCAACTTGATTGTATACAGTGACGACTATTTATCCCTAATGTCTACTTCCGGTCAATCCATCTACGTTAACCGAGGTGGCTATGTGTa                                                                                                          <  1:360415/300‑1 (MQ=255)`  
                                                                                                                                                                                                 `cGATATGAACGTTGAGACTGCCGCTGAGTTATCAGCTGTGAACGACATTCTGGCGTCTATCGGTGAACCTCCGGTATCAACGCTGGAAGGTGACGCTAACGCAGATGTAGCGAACGCTCGGCGTATTCTCAACAAGATTAACCGACAGATTCAATCTCGTGGATGGACGTTCAACATTGAGGAAGGCATAACGCTACTACCTGATGTTTACTCCAACCTGATTGTATACAGTGACGACTATTTATCCCTAATGTCTACTTCCGGTCAATCCATCTACGTTCACCGCGGTGGCTATGTGTAt                                                                                                         >  1:355294/1‑301 (MQ=255)`  
                                                                                                                                                                                                  `gATATGAACGTTGAGACTGCCGCTGAGTTATCAGCTGTGAACGACATTCTGGCGTCTATCGGTGAACCTCCGGTATCAACGCTGGAAGGTGACGCTAACGCAGATTCAGCGAACGCTCGGCGTATTCTCAACAAGATTAACCGACAGATTGAATCTCGTGGATGGACGTTCAACATTGAGGAAGGCATAACGCTACTACCTGATGTTTACTCCAACCTGATTGTATACAGTGACGACTATTTATCCCTAATGTCAACTTCCTGTCAATCCATCTACGTTAACCGAGGTggactgtctctat                                                                                                        >  1:80892/1‑290 (MQ=255)`  
                                                                                                                                                                                                  `gATATGAACGTTGAGACTGCCGCTGAGTTATCAGCTGTGAACGACATTCTGGCGTCTATCGGTGAACCTCCGGTATCAACGCTGGAAGGTGACGCTAACGCAGATGCAGCGAACGCTCGGCGTATTCTCAACAAGATTAACCGACATATTCAATCTCGTGGATGGACGTTCAACATTGAGGAAGGCATAACGCTACTACCTGATGTTTACTCCAACCTGATTGTATACAGTGACGACTATTTATCCCTAATGTCTACTTCCGGTCACTCCATCTACGTTAACCGAGGTGGcctgtctctta                                                                                                        >  1:100304/1‑291 (MQ=255)`  
                                                                                                                                                                                                   `atatGAACGTTGAGACTGCCGCTGAGTTATCAGCTGTGAACGACATTCTGGCGTCTATCGGTGAACCTCCGGTATCAACGCTGGAAGGTGACGCTAACGCAGATGTAGCGAACGCTCGGCGTATTATCAACAAGATTAACCGACAGATTCAATCTCGTGGATGGACGTTCAACATTGAGGAAGGCATAACGCTACTACCTGATGTTTACTCCAACCTGATTGTATACAGTGACGACTATTTATCACTAATGTCTACTTCCGGTCAATCCATCTACGTTAACCGAGGTGGCTATGTGTATGa                                                                                                       >  1:452212/1‑301 (MQ=255)`  
                                                                                                                                                                                                   `atatGAACGTTGAGACTGCCGCTGAGTTATCAGCTGTGAACGACATTCTGGCGTCTATCGGTGAACCTCCGGTATCAACGCTGGAAGGTGACGCTAACGCAGATGCAGCGAACGCTCGGCGTATTCTCAACAAGATTAACCGACAGATTCAATCTCGTGGATGGACGTTCAACATTGAGGAAGGCATAACGCTACTACCTGATGTTTACTCCAACCTGATTGTATACAGTGACGACTATTTATCCCTAATGTCTACTTCCGGTCAATCCATCTACGTTAACCGAGGTGGCTATGTGTATgt                                                                                                       >  1:263614/1‑300 (MQ=255)`  
                                                                                                                                                                                                   `atatGAACGTTGAGACTGCCGCTGAGTTATCAGCTGTGAACGACATTCTGGCGTCTATCGGTGAACCTCCGGTATCAACGCTGGAAGGTGACGCTAACGCAGACGTAGCGAACGCTCGGCGTATTCTCAACAAGATTAACCGACAGATTCAATCTCGTGGATGGACGTTCAACATTGAGGAAGGCATAACTCTACTACCTGATGTTTACTCCAACCTTATTGTATACAGTGACGACTATTTATCCCTAATGTCTACTTCCGGTCAATCCATCTACGTTAACCGAGGTGGCTATGTGTATGa                                                                                                       >  1:8557/1‑301 (MQ=255)`  
                                                                                                                                                                                                    `tatGAACGTTGAGACTGCCGCTGAGTTATCAGCTGTGAACGACATTCTGGCGTCTATCGGTGAATCTCCGGTATCAACGCTGTAAGGTGACGCTAACGCAGATGTAGCGAACGCTCGGCGTATTCTCAACAAGATTAACCGACAGATTCAATCTCGTGGATGGACGTTCAACATTGAGGAAGGCATAACGCTACTACCTGATGTTTACTCCAACCTGATTGTATACAGTGACGACTATTTATCCCTAATGTCTACTTCCGGTCAATCCATCTACGTTAACCGAGGTGGCTATGTGTATGAc                                                                                                      <  1:13433/301‑1 (MQ=255)`  
                                                                                                                                                                                                    `tatGAACGTTGAGACTGCCGCTGAGTTATCAGCTGTGAACGACATTCTGGCGTCTATCGGTGAACCTCCGGTATCAACGCTGGAAGGTGACGTTAACGCAGATGTAGCGAACGCTCGGCGTATTCTCAACAAGATTAACCGACAGATTCAATCTCGTGGATGGACGTTCAACATTGAGGAAGGCATAACGCTACTACCTGATGTATACTCCAACCTGATTGTATACAGTGACGACTATTTATCCCTAATGTCTACTTCCGGTCAATCCATCTACGTTAACCGAGGTGGCTATGTGTATGAc                                                                                                      <  1:413946/301‑1 (MQ=255)`  
                                                                                                                                                                                                    `tatGAACGTTGAGACTGCCGCTGAGTTATCAGCTGTGAACGACATTCTGGCGTCTATCGGTGAACCTCCGGTATCAACGCTGGAAGGTGACGCTAACGCAGATGTAGCGAACGATCGGCGTATTCTCAACAAGATTAACCGACAGATTCAATCTCGTGGATGGACGTTCAACATTGAGGAAGGCATAACGCTACTACCTGATGTTTACTCCAACCTGATTGTATACAGTGACGACTATTTATCCATAATGTCTACTTCCGGTCAATCCATCTACGTTAACCGAGGTGGCTATGTGTATGAc                                                                                                      <  1:38898/301‑1 (MQ=255)`  
                                                                                                                                                                                                    `aatGAACGTTGAGACTGCCGCTGAGTTATCAGCTGTGAACGACATTCTGGCGTCTATCGGTGAACCTTCGGTATCAACGCTGGAAGGTGACGCTAACGCAGATGCAGCGAACGCTCGGCGTATTCTCAACAAGATTAACCGACATATTCAATCTCGTGGATGGACGTTCAACATTGAGGAAGGCATAACGCTACTACCTGATGTTTACTCCAACCTGATTGTATACAGTGACGACTATTTATCCCTAATGTCTACGTCCGGTCAATCCATCTACGTTAACCGAGGTGGCTATGTGTATGAc                                                                                                      <  1:349729/300‑1 (MQ=255)`  
                                                                                                                                                                                                     `atGAACGTTGAGACTGCCGCTGAGTTATCAGCTGTGAACGACATTCTGGCGTCTATCGGTGAACCTCCGGTATCAACGCTGGAAGGTGACGCTAACGCAGATGTAGCGAACGCTCGGCGTATTCTAAACAAGATTAACCGACAGATTCAATCTCGTGGATGGACGTTCAACATTGAGGAAGGCATAACGCTACTACCTGATGTTTACTACAACCTGATTGTATACAGTGACGACTATTTATCCCTAATGTCTACTTCCGGTCAATAAATCTACGTTAACCGAGGTGGCTATGTATATGAcc                                                                                                     >  1:47371/1‑301 (MQ=255)`  
                                                                                                                                                                                                                       `gCTGAGTTATCAGCTGTGAACTACATTCTGGCGTCTATCGGTGAACCTCCGGTATCAACGCTGGAAGGTGACGCTAACGCAGATGTAGCGAACGCTCGGCGTATTCTCAACAAGATTAACCGACAGATTCAATCTCGTGGATGGACGTTCAACATTGAGGAAGGCATAACGCTACTACGTGATGTTTACTCCAACCTGATTGTATACAGTGACGACTATTTATCCCTAATGTCTACTTCCGGTCAATCAATCTACGTTAACCGAGGTGGCTATGTGTATGACCGAACGAGTCCATCAGAcc                                                                                   >  1:61240/1‑301 (MQ=255)`  
                                                                                                                                                                                                                                `tcacccttgAACGACATTCTGGCGTCTATCGGTGATCCTCCTGTATCAACGCTGGAAGGTGACGCTAACGCAGATGTAGCGATCTCTCGGCGTATTCTCAACAAGATTAACCGACAGATTCAATCTCGTGGATGGACGTTCAACATTGAGGAAGGCATAACTCTACTACCTGATGTTTACTCCAACCTGATTGTATACAGTGACGACTATTTATCCCTAATGTCTACTTCCGGTCAATCCATCTACGTTAACCGAGGTGGCTATGTGTATGACCGAACGAGTCAATCAGACCGCTTTGAct                                                                          <  1:280678/294‑1 (MQ=255)`  
                                                                                                                                                                                                                                 `cAGCTGTTAACGACATTCTGGCGTCTATCGGTGAACCTCCGGTATCAACGCTGGAAGGTGACGCTAACGCAGATGTAGCGAACGCTCGGCGTATTCTCAACAAGATTAACCGACAGATTCAATCTCGTGGATGGACGTTCAACATTGAGGAAGGCATAACGCTACTACCTGATGTTTACTCCAACCTGATTGTATACAGTGACGACTATTTATCCCTAATGTCTACTTCCGGTCAATCCATCTACGTTAACCGAGGTGGCTATGTGTATGACCGAACGAGTCAATCAGACCGCTTTGActc                                                                         <  1:39312/301‑1 (MQ=255)`  
                                                                                                                                                                                                                                     `tgtgAACGACATTCTGGCGTCTATCGGTGAACCTCAGGTATCAACGCTGGAAGGTGACGCTAACGCAGATGCAGCGAACGCTCGGCGTATTCTAAACAAGATTAACCGACATATTCAATCTCGTGGATGGACGTTCAACATTGAGGAAGGCATAACGCTACTACCTGATGTTTACTCCAACCTGATTGTATACAGTGACGACTATTTATCCCTAATGTCTACTTCCGGTCAATCCATCTACGTTAACCGAGGTGGCTATGTGTATGACCGAACGAGTCAATCAGACCGCTTTGACTCTGGt                                                                     <  1:460109/301‑1 (MQ=255)`  
                                                                                                                                                                                                                                      `gtgAACGACATTCTGGCGTCTATCGGTGAACCTCCGGTATCAACGCTGGAAGGTGACGCTAACGCAGATGTAGCTAACGCTCGGCGTATTCTCAACAAGATTAACCGACAGATTCAATCTCGTGGATGGACGTTCAACATGGAGTAAGGCATAACGCTACTACCCGATGTTTACTCCAACCTGATTGTATACAGTGACGACTATTTATCCCTAATGTCTACTTCCGGTCAATCCATCTACGTTAACCGAGGTGGCTATGTGTATGACCGAACGAGCCAATCAGACCGCTTTGACTCTGGtt                                                                    >  1:378263/1‑300 (MQ=255)`  
                                                                                                                                                                                                                                          `gtataagagacaggTCTATCGGGGAACCTCCGGTAGCAACGCTGGAAGGTGACGCTAACGCAGATGTAGCGAACGCTCGGCGTATTCTCAACAAGATTAACCGACAGATTCAATCGCGTGGATGGACGTTCAACATTGAGGAAGGCATAACGCTACTACCTGATGTTTACTCCAACCTGATTGTATACGGTGACGACTATTTATCCCTAATGTCTACTTCCGGTCAATCCATCTACGTTAACCGAGGTGGCTATGTGTATGACCGAACGAGTCAATCAGACCGCTTTGACTCTGGTATTAc                                                                <  1:329955/288‑1 (MQ=255)`  
                                                                                                                                                                                                                                          `atgtgtataagagacagaTCGGTGAACCTCCGGTATCAACGCTGGAAGGTGACGCTAACGCAGATGTAGCGAACGCTCGGCGTATTCTCAACAAGATTAACCGACAGATTCAATCTCGTGGATGGACGTTCAACATTGAGGAAGGCATAACGCTACTACCTGATGTTTACTCCAACCTGATTGTATACAGTGACGACTATTTATCCCTAATGTCTACTTCCGGTCAATCCATCTACGTTAACCGAGGTGGCTATGTGTATGACCGAACGAGTCAATCAGACCGCTTTGACTCTGGTATTAc                                                                <  1:113556/284‑1 (MQ=255)`  
                                                                                                                                                                                                                                          `aCGACATTCGGGCGTCTATCGGTGAACCTCCGGTATCAACGCGGGAAGGTGACGCTAACGCAGATGTAGCGAACGCTCGGCGTATTCTCAACAAGATTAACCGACAGATTCAATCTCGTGGATGGACGTTCAACATTGAGGAAGGCATAACGCTACTACCTGATGTTTACTCCAACCTGATTGTATACAGTGACGACTATTTATCCCTAATGTCTACTTCCGGTCAATCCATCTACGTTAACCGAGGTGGCTATGTGTATGACCGAACGAGTCAATCAGACCGCTTTGACTCTGGTATTAc                                                                <  1:138548/301‑1 (MQ=255)`  
                                                                                                                                                                                                                                                    `ggCGTCTATCGGTGAACCTCCGGTATCAACGCTGGAAGGTGACGCTAACGCAGATGTAGCGAACGCTCGGCGTATTCTCAACAAGATTAACCGACAGATTCAATCTCGTGGATGGACGTTCAACATTGAGGAAGGCATAACGCTACTACCTGATGTTTACTCCAACCTGATTGTATACAGTGACGACTATTTATCCCTAACGTCTACTTCCGGTCAATCCATCTACGTTAACCGAGGTGTCTATGTGTATGACCGAACGAATAAATCAGACCGCTTTGAatcctgtctcttatacacatct                                                      >  1:189922/1‑282 (MQ=255)`  
                                                                                                                                                                                                                                                       `gTCTATCGGTGAACCGCCGGTATCAACGCTGGAAGGTGACGCTAACGCAGATGTAGCGAACGCTCGGCGTATTCTCAACAAGATTAACCGACAGATTCAATCTCGTGGATGGACGTTCAACATTGAGGAAGGCATAACGCTACTACCTGATGTTTACTCCAACCTGATTGTATACAGTGACGACTATTTATCCCTAATGTCTACTTCCGGTCAATCCATCTACG‑TAACCGAGGTGGCTATGTGTATGACCGAACGAGTCAATCAGACCGCTTTGACTCTGGTATTACTGTGAACATTATTc                                                  <  1:438501/301‑1 (MQ=255)`  
                                                                                                                                                                                                                                                           `ttCGGTGACCCTCGGGTATCAACGCGTGAAGGTCACGCTAACGCAGATGTAGCGAACGCGCGGCGTATTCTCAACAAGATTAACCGCCAGATTCCATCTCGTGGATGGACGTTCAACATTGAGGAAGGCATAACGCTACTACCTGATGTTTACTCCAACCTGATTGGATACAGTGACGGCTATTTATCCCTAATGTCTACTTCCGGTCAATCCATCTACGTTAACCGAGGTGGCTATGTGTATGACCGAACGAGTCAATCAGACCGCTTTGACTCTGGTATTACTGTGAACATTATTCGtc                                               <  1:433307/300‑1 (MQ=255)`  
                                                                                                                                                                                                                                                                        `cGGTATCAAAGCTGGAAGGTGACGCTAACGCAGATGTAGCGAACGCTCGGCGTATTATCAACAAGATTAACCGACAGATTCAATCTAGTGGATGGACGTTCAACATTGAGGAAGGCATAACGCTACTACCTGATGTTTACTCCAACCTGATTGTATACAGTGACGACTATTTATCCCTAATGTCTACTTCCGGTCAATCCATCTACGTTAACCGAGGTGGCTATGTGTATGACCGAACGAGTCAATCAGACCGCTTTGACTCTGGTATTACTGTGAACATTATTCGTCTCCGCGACTACGa                                  <  1:447985/301‑1 (MQ=255)`  
                                                                                                                                                                                                                                                                          `gTATCAATGCTGGAAGGTGACGCTAAAGCAGATGCAGCGAACGCTCGGCGTATTCTCAACAAGATTAACCGACATATTCAAGCTCGTGGATGGACGTTCAACATTGAGGAAAGCATAACACTACTACCTGATGTTTACTCCAACCTGATTGTATACAGTGACGACTATTTATCCCTAATGTCTACTTCCGGCCAATCCATCTACTTTAACCGTGGTGTCTATGTGTATGCCCGTACTAGTCAATCAGACCTCTTTTACTCTGGTAGTACTGTGAACATTATTCGTCTCCGCGACTACGATg                                <  1:76887/301‑1 (MQ=255)`  
                                                                                                                                                                                                                                                                          `gTATCAACGCTGGAAGGTGACGCTAACGCAGATGCCGCGAACGCTCGGCGTATTCTCAACAAGATTAACCGACATATTCAATCACGTGGATGGACGTTCAACATTGAGGAAGGCATAACGCTACTACCTGATGTTTACTCCAACCTGATTGTATACAGTGACGACAATTTATCACTAATGTCTACTTCCGGTCAATCCATCTACGTTAACCGAGATGGCTATGTGTATGACCGCACGAGTCCATCAGAACGCTTTAACTCTGGTATTACTATGAACATTAGTCGTCTCCGCGACTACGAtt                                >  1:315434/1‑300 (MQ=255)`  
                                                                                                                                                                                                                                                                                `aCGCTGGAAGGTGACGCTAACGCAGATGCAGCGAACGCTCGGCGTATTCTCAACAAGATTAACCGACATATTCAATCTCGTGGATGGACGTTCAACATTGAGGAAGGCATAACGCTACTACCTGATGTTTACTCCAACCTGATTGTATACAGTGACGACTATTTATCCCTAATGTCTACTTCCGGTCAATCCATCTACGTTAACCGAGGTGGCTATGTGTATGACCGAACGAGTCAATCAGACCGCTTTGACTCTGGTATTACTGTGAACATTATTCGTCTCCGCGACTACGATGAGATGc                          >  1:5078/1‑301 (MQ=255)`  
                                                                                                                                                                                                                                                                                      `gAAGGTGACGCTAACGCAGATGTAGCGAACGCTCGGCGTATTCTCAACAAGATTAACCGACAGATTCAATCTCGTGGATGGACGTTCAACATTGAGGAAGGCATAACGCTACTACCTGATGTTTACTCCAACCTGATTGTATACAGTGACGACTATTTATCCCTAATGTCTACTTCCGGTCAATCCATCTACGTTAACCGAGGTGGCTATGTGTATGACCGAACGAGTCAATCAGACCGCTTTGACTCTGGTATTACTGTGAACATTATTCGTCTCCGCGACTACGATAAGATGCCTGAgg                    >  1:222601/1‑300 (MQ=255)`  
                                                                                                                                                                                                                                                                                        `aGGTGACGCTAACGCAGATGTAGCGAACGCTCGGCGTATTCTCAACAAGATTAACCGACAGATTCAATCTCGTGGATGGACGTTCAACATTGAGGAAGGCATAACGCTACTACCTGATGTTTACTCCAACCTGATTGTATACAGTGACGACTATTTATCCCTAATGTCTACTTCCGATCAATCCATCTACGTTAACCGAGGTGGCTATGTGTATGACCGAACGAGTCAATCAGACCGCTTTGACTCTGGTATTACTGTGAACATTATTCGTCTCCGCGACTACGATGAGATGCCTGAGTGc                  <  1:386036/301‑1 (MQ=255)`  
                                                                                                                                                                                                                                                                                                    `cGCAGATGCAGCGAACGCTCGGCGTATTCTCAACAAGATTAACCGACATATTCAATCTCGTGGATGGACGTTCAACATTGAGGAAGGCATAACGCTACTACCTGATGTTTACTCCAACCTGATTGTATACAGTGACGACTATTTATCCCTAATGTCTACTTCCGGTCAATCCATCTACGTTAACCGAGGTGGCTATGTGTATGACCGAACGAGTCAATCAGACCGCTTTGACTCTGGTATTACTGTGAACATTATTCGTCTCCGCGACTACGATGAGATGCCTGAGTGCTTCCGTTACTgg      <  1:214828/301‑1 (MQ=255)`  

                                                                                                                                                                                                                                                                                                            `|`                                                                                                                                                                                                                                                                                                          
`GCGAGTGTTACTTCAACAGAAGAAACCTTAACGCCAGCACAGGAGGCCGCACGCACCCGCGCTGCTAACAAAGCCCGAAAGGAAGCTGAGTTGGCTGCTGCCACCGCTGAGCAATAACTAGCATAACCCCTTGGGGCCTCTAAACGGGTCTTGAGGGGTTTTTTGCTGAAAGGAGGAACTATATGCGCTCATACGATATGAACGTTGAGACTGCCGCTGAGTTATCAGCTGTGAACGACATTCTGGCGTCTATCGGTGAACCTCCGGTATCAACGCTGGAAGGTGACGCTAACGCAGATGCAGCGAACGCTCGGCGTATTCTCAACAAGATTAACCGACAGATTCAATCTCGTGGATGGACGTTCAACATTGAGGAAGGCATAACGCTACTACCTGATGTTTACTCCAACCTGATTGTATACAGTGACGACTATTTATCCCTAATGTCTACTTCCGGTCAATCCATCTACGTTAACCGAGGTGGCTATGTGTATGACCGAACGAGTCAATCAGACCGCTTTGACTCTGGTATTACTGTGAACATTATTCGTCTCCGCGACTACGATGAGATGCCTGAGTGCTTCCGTTACTGGATTG  >  NC_001604.1/24046‑24642`

  

Alignment Legend

Aligned base mismatch/match (shaded by quality score): `ATCG/ATCG` `< 3 ≤` `ATCG/ATCG` `< 9 ≤` `ATCG/ATCG` `< 16 ≤` `ATCG/ATCG` `< 31 ≤` `ATCG/ATCG` `< 38 ≤` `ATCG/ATCG`

Unaligned base: `atcg`    Masked matching base: `atcg`    Alignment gap: `‑`    Deleted base: `‑`
